# Range‐Wide Camera Trapping for the Australian Cassowary Reveals Habitat Associations With Rainfall and Forest Quality

**DOI:** 10.1002/ece3.71464

**Published:** 2025-06-04

**Authors:** Skye Elise Anderson, Zachary Amir, Tom Bruce, Matthew Scott Luskin

**Affiliations:** ^1^ The Wildlife Observatory of Australia (WildObs) QCIF Brisbane Queensland Australia; ^2^ School of the Environment & Centre for Biodiversity and Conservation Science University of Queensland Brisbane Queensland Australia; ^3^ Terrestrial Ecosystem Research Network University of Queensland Brisbane Queensland Australia; ^4^ Centre for Biodiversity and Conservation Science University of Queensland Brisbane Queensland Australia

**Keywords:** abiotic factors, anthropogenic disturbances, bird, conservation, detectability, hierarchical modelling, interactive effects, occupancy, species distribution modelling

## Abstract

The Australian Wet Tropics rainforests are a biodiversity hotspot covering just 0.2% of the continent's land area. However, historic forest loss, modern fragmentation, and climate change continue to threaten these ecosystems. Southern cassowaries ( *Casuarius casuarius* ) are large flightless birds restricted to closed‐canopy tropical forests in Australia. Cassowaries are obligate frugivores whose dispersal of large‐seeded plants is considered a keystone species interaction supporting forest regeneration. We conducted camera trapping across cassowaries' Australian range and quantified habitat associations using hierarchical models that account for imperfect detection. Cassowary detections were significantly higher in rainforests compared to adjacent wet sclerophyll closed‐canopy forests, confirming their status as habitat specialists. Cassowaries' relative abundance (λ in Royle‐Nichols modelling) declined with forest degradation and rainfall but was not strongly affected by human footprint or elevation. This aligns with observations of them occasionally foraging on anthropogenic food sources at the edges of large intact forests (e.g., where there are human‐planted fruit trees). These findings provide the ecological reasons underpinning known cassowary hotspots in large rainforests that are relatively dry. It would be valuable to deepen our understanding of their persistence in degraded rainforests near humans via diet and survival studies, and we caution that their association with rainfall means that they may be impacted by climate change.

## Graphical abstract

We conducted large‐scale camera trapping across the Australian range of the southern cassowary and quantified habitat associations. Cassowary relative abundance peaked in intact rainforest areas with comparatively less rainfall, but also showed weak avoidance of humans. These findings expand on the existing research identifying particular areas with high densities by revealing the ecological drivers underpinning these pockets of conservation success.

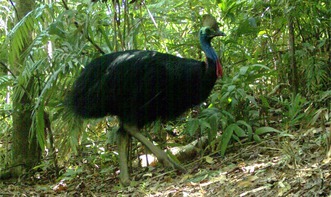

## 1 Introduction

Australia's tropical rainforests cover 0.2% of land area but are home to 50% of plant species and 35%–40% of mammal and bird species in Australia (Clarke et al. 2007). This biodiversity hotspot (Myers et al. 2000) suffered historical deforestation, whose legacy incurs contemporary effects of fragmentation and edges, exacerbating the impact of invasive species and climate change (Stork et al. 2011). Degraded tropical forests experience shifts in wildlife composition, replacing sensitive interior species with disturbance‐tolerant generalists, especially where they have access to novel anthropogenic food subsidies (Luskin et al. 2017, 2023). The loss of large vertebrates and their functions in forest ecology and regeneration, such as seed dispersal, is particularly deleterious (Dirzo et al. 2014; Gardner et al. 2019). Shifts in species assemblages can also result in a deficit of important ecological interactions and services, hindering restoration. Therefore, retaining megafaunal communities is a key component of conserving forest composition and function.

**FIGURE 1:**
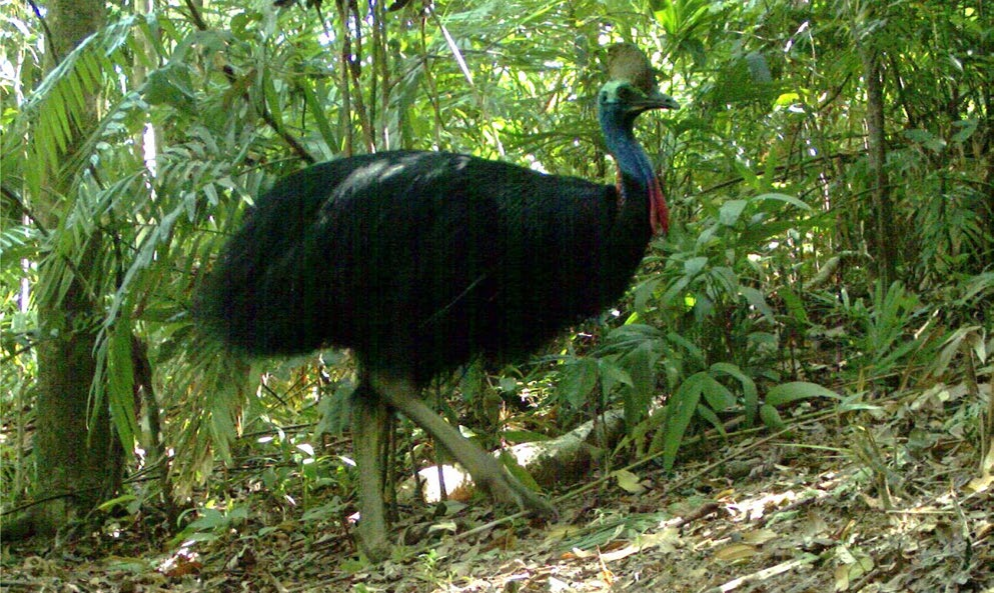
Camera trap photo of a cassowary in Daintree National Park from a bush camera (as opposed to a road camera).

The southern cassowary (*Casuarius casuarius*; hereafter, just cassowary) inhabits the tropical forests of northeast Australia. At 50‐75 kg, they are the third largest bird in the world (Bertram 2014; Campbell et al. 2015). Cassowaries play a crucial role in the region's tourism, partly due to their striking appearance, characterised by a vividly coloured head and neck, a prominent casque, and shaggy black plumage (Figure 1). Cassowaries are also culturally significant to Indigenous groups and the wider communities of the Australian tropical forests (Hill et al. 2010; Andy 2021; State of the Wet Tropics 2022–2023).

Cassowary conservation is inextricably linked to Australia's tropical forest ecology and health. In most tropical forests, more than 70% of woody plants produce vertebrate‐dispersed seeds (Gentry 1982; Malhi et al. 2014), and, in general, the dispersal services provided by large vertebrates cannot be substituted by other smaller species (Vanthomme et al. 2010; Campos‐Arceiz et al. 2012). Cassowaries eat mostly fleshy fruits of a wide range of sizes from more than 1500 species of plants (Bradford et al. 2008), including very large seeds, for which they provide unique dispersal opportunities (Noble 1991; Dehaudt, Bruce, et al. 2024). In fact, they are the only extant dispersers of the largest‐seeded plants in Australia's tropical forests and fulfill ecological roles similar to large frugivorous birds and mammals in other tropical forests that have received more research attention, such as the hornbills and elephants of Asian and African tropical forests (Cochrane 2003; Forget et al. 2007; Bradford and Westcott 2010). Cassowaries are also highly mobile, dispersing seeds up to 1.5 km from the parent trees (Westcott et al. 2005), thereby facilitating the movement of native plants between tropical forest patches and playing an important role in degraded forest plant community regeneration (Campbell et al. 2023). On the contrary, disturbance‐tolerant invasive species that thrive in edge habitats, such as feral pigs ( *Sus scrofa* ), primarily crush and kill seeds, preventing them from germinating (Pedrosa et al. 2019).

Although cassowaries are a large charismatic megafauna and maintain ecologically important roles in tropical forests, there is little robust information on their habitat associations (Dennis 2024). After European settlement in the 1870s, one‐quarter of the tropical forest in northeast Australia was cleared, particularly on the lowlands, which is thought to be ideal cassowary habitat (Erskine 2002; State of the Wet Tropics 2022–2023). Cassowary populations declined throughout the 1900s due to habitat loss (Crome and Moore 1990). Climate change is also rapidly altering Australia's tropical forests, with regional warming and increased heatwaves resulting in declining abundances of possums and bird species (Williams and de la Fuente 2021; de la Fuente and Williams 2023). Early ecological observations by Crome (1976) noted that fruit scarcity during the wet season likely limits cassowary populations in Australian tropical forests. Prior work suggested cassowary densities may peak at lower elevations (< 750 m asl), yet this is not consistent (Westcott et al. 2014). One study that tracked the movements of individuals demonstrated that cassowaries maintained core home ranges in remnant tropical forests but frequently ventured into fragmented forest patches, pastoral lands, and residential areas (Campbell et al. 2012), and cassowaries in modified landscapes are often observed consuming cultivated fruits (Crome 1976; Westcott et al. 2005; Sasaoka and Ohtsuka 2010). Taken together, there are inconsistencies in habitat associations, degree of interior forest specialisation, and the extent to which they use forest edges.

We conducted large‐scale camera trapping across the cassowary range in tropical rainforests and adjacent sclerophyll habitats. We used Royle–Nichols hierarchical models to assess variation in relative abundance (λ) with covariates and to predict cassowary relative abundance across their range. For these predictive maps, we used a model selection process to choose the most parsimonious set of variables hypothesised to influence cassowary relative abundance. In terms of general habitat associations, we hypothesised that cassowary relative abundance would be negatively associated with forest degradation and humans because the species is considered an interior forest specialist (Westcott et al. 2014). More specifically, we predicted that cassowary relative abundance would have: (1) a positive relationship with intact forests as seen in other forest specialists in the region (Heise‐Pavlov et al. 2014), and we measured forest quality using the Forest Landscape Integrity Index (Grantham et al. 2020); (2) a negative relationship with humans and their associated disturbances, as measured by Human Footprint Index (Venter et al. 2016); (3) an inverse relationship with elevation as suggested by some prior work (Westcott et al. 2014); and (4) a negative relationship with rainfall as wetter periods may correspond with decreased fruit availability.

## 2 Methods

### 2.1 Study Landscapes

We conducted camera trap surveys across the southern cassowary range in the tropical forests of northeast Australia. This region, designated as the Wet Tropics of Queensland, was protected as a UNESCO World Heritage site in 1988 and covers approximately 894,420 ha of predominantly tropical forest (UNESCO World Heritage Centre 2023). Following its designation, tree clearing and logging were largely halted (Crome et al. 1992). Over 87% of the Wet Tropics forested area is protected as national parks, conservation parks, state forests, and forest reserves.

We collected data from 10 landscapes, where each “landscape” refers to a distinct survey area of 11.6 to 1200 km^2^, with cameras from one survey area and time separated from other survey areas at the same time by at least 10 km, and typically encompassing a single national park or multiple nearby national parks (Table 1). Our surveys spanned an elevational range from 2 to 1200 m asl, predominantly within tropical forest habitats, with some sclerophyll forest along the peripheries. Wooroonooran was the largest landscape and was split to be comparable with the other landscapes (Wooroonooran NP core and Wooroonooran‐Goldfield). Similarly, the nearby small forest fragments of Eacham, Crater Lake and Curtain Fig National Park were combined into one landscape due to their close proximity.

**TABLE 1:** Study site descriptions.

Landscape	Forest size (km^2^)	Elevation (m)	Monthly rainfall (mm)	Forest integrity	Human footprint
Daintree NP	1200	61	253	7.7	22.2
Wooroonooran NP core	798	648	252	7.8	3.6
^X^Paluma Range NP	763.7	773	155	8.7	4.1
Wooroonooran Goldfield	361	180	289	8.6	2
Koombooloomba‐Tully Falls NPs	292.6	803	150	7.9	5.9
Mt Lewis NP	278.6	986	140	9.8	0.1
Kirrama NP	172.9	567	139	7.5	7.4
Danbulla NP	120	755	124	7	2
^X^Tumoulin NP^a^	18.82	1022	116	6.9	2.8
^X^Eacham‐Curtain Fig^a^	11.6	745	146	1.2	10.5

*Note:* Contiguous forest size was obtained from Queensland Government (2024). For all other covariates, we display the mean value of the 3 km^2^ sampling units that were sampled at each landscape (Figure 2). Forest integrity is the Forest Landscape Integrity Index (0–10; Grantham et al. 2020), and human footprint is the Human Footprint Index (0–50; Venter et al. 2016). ^X^denotes sites without cassowary detections.

Abbreviation: NP, National park.

^a^ Indicates the survey was not included in the RN modelling reported in the main text due to no detections and unsuitable habitat. Eacham–Curtain Fig landscape includes areas of Crater Lakes NP.

### 2.2 Camera Trapping

We deployed 454 cameras at 240 different sites across two distinct field campaigns spanning April 2019—October 2020 and September 2022—March 2023 (Table 2). Our dataset follows a hierarchical structure, where two cameras were deployed at each site, one on a linear feature in the environment (i.e., a road or trail) and another in the ‘bush’, 50–75 m into the forest. This was done because trail use by cassowaries varies (Crome and Moore 1990; Westcott 1999), and detectability on trail versus bush cameras was explicitly accounted for in the detection formula of our hierarchical modelling.

**TABLE 2:** Survey details and results.

Survey ID	Cams	Trap nights	Start date	End date	Captures	RAI	Road cams	Bush cams
Wooroonooran core ‐ 2022	66	4203	2022‐12‐20	2023‐03‐19	293	6.9	34	32
Daintree	44	2666	2022‐09‐20	2022‐12‐07	180	6.1	23	21
Wooroonooran core ‐ 2019	33	1384	2019‐04‐16	2019‐05‐30	18	1.3	18	15
Koombooloomba‐ Tully Falls	36	1250	2019‐09‐02	2019‐10‐23	14	1.1	18	18
Wooroonooran‐Goldfield	48	3223	2022‐12‐12	2023‐03‐17	33	1	24	24
Mt Lewis	44	2640	2022‐09‐27	2022‐12‐15	15	0.5	22	22
Danbulla	42	2520	2022‐10‐04	2023‐01‐05	10	0.3	22	20
Kirrama	34	1619	2019‐07‐17	2019‐09‐05	3	0.2	18	16
^X^Paluma Range	44	3342	2019‐11‐05	2020‐02‐05	0	0	24	20
^X^Eacham ‐ Curtain Fig^a^	32	1826	2022‐12‐15	2023‐03‐07	0	0	16	16
^X^Tumoulin^a^	32	1407	2019‐04‐12	2019‐05‐28	0	0	17	15

*Note:* The number of cameras deployed (cams) and the relative abundance index (RAI) measure the number of cassowaries detected per 100 trap nights, where we considered cassowary detections independent if they occurred at least 30 min apart. ^X^denotes sites without cassowary detections. Eacham/Curtain Fig and Tumoulin were excluded from the hierarchical modelling.

^a^ Indicates the survey was not included in the RN modelling reported in the main text.

We standardised camera deployment between landscapes by attaching cameras to trees 0.2 to 0.3 m high and spacing our sampling locations 1–2.2 km apart from each other (mean = 912.9 m, SD = 564.6 m). Sampling sites were closer together (~450 m) in the smaller forest patches of Eacham, Crater Lakes and Curtain Fig, with this variation being explicitly accounted for in our spatial resampling process described later. Cameras were active for 1 to 93 days (mean = 57, SD = 23) at each site, with 26,014 total trap nights across the entire study (Table 2). Four camera brands were used: Bushnell (*n* = 200 placements), Reconyx (*n* = 161), Browning (*n* = 66), and Hawkray (*n* = 52).

### 2.3 Data Preparation

**FIGURE 2:**
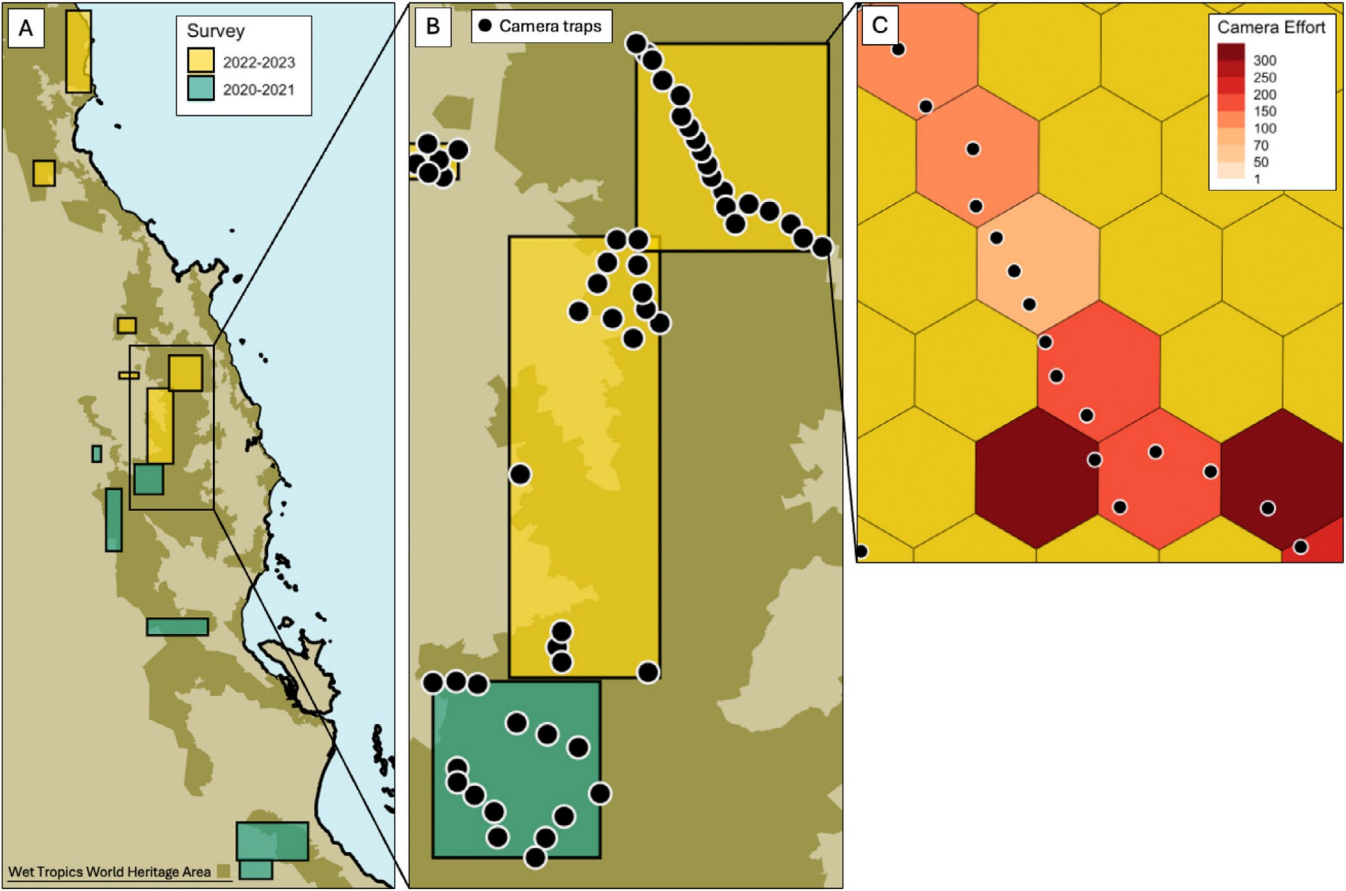
(A) Map showing the study area in Northeast Queensland with bounding boxes indicating sampled landscapes. (B) Inset of the Atherton tablelands where much sampling took place; each point represents a site where two cameras were deployed at paired trail and bush sites. (C) Inset of Wooroonooran National Park, showing spatially resampled camera deployments into 3 km^2^ hexagonal sampling units that are colour‐coded by camera effort in trap nights.

We collated all cameras into a standardised dataset to analyse within a single analytical framework. To conform to the population closure assumption of single‐season hierarchical models, we ensured the sampling duration of each camera was no more than 93 days, within which the population size is assumed to be stable. This is reasonable for southern cassowaries, whose lifespan exceeds 40 years and generation time is 17–20 years (Birdlife 2017). To conform to the spatial independence assumption despite variable distances between sampling sites, we spatially resampled all cameras into 3 km^2^ sampling units that are nested in the broader landscapes (Figure 2). This spatial grain was chosen to be larger than the average home range of a female cassowary, reported as 2.06 km^2^ by Moore (2007) and 0.71 km^2^ by Campbell et al. (2012). We used a 5‐day window as the sampling occasion to decrease the number of false zeros in the dataset (i.e., sampling occasions with no detections) and increase detection probabilities (Brodie et al. 2018). Finally, we produced a detection history matrix containing presence‐absence data across all sampling units (i.e., rows) by the 5‐day window (i.e., columns).

### 2.4 Generating Covariates

We incorporated six key covariates into our modelling that could influence cassowary detection probability across the study region. We included the number of cameras active per sampling unit to account for variation in the sampling effort (observation unit‐level covariate). Additionally, we incorporated trail status to determine the likelihood of animals being detected on linear features or in the bush (observation‐level). We also tested a more detailed feature type to more finely discriminate whether the detectability varied among hiking trails, dirt roads, paved roads, or in the bush (observation‐level). We tested camera brand as a covariate (observation‐level) and whether detectability varied among the two deployment teams that collected data (observation‐level). Finally, we assessed if there were temporal variations in detectability by classifying observation windows into one of four seasons: shoulder‐wet (October—November), wet (December—March), shoulder‐dry (April—May) and dry (June—September). Full model selection for all the detection covariates is not included in the main text (Table S1).

We included four environmental and human disturbance covariates in cassowary relative abundance state formula. We generated our spatial covariates using spatial raster layers or shape files with a 3 km^2^ buffer that grouped cameras into sampling units using the terra package in R (Hijmans 2024). The environmental habitat covariates in our analysis were elevation, the 3‐s SRTM Derived Digital Elevation Model in metres (Geoscience Australia 2010), and average monthly rainfall, taken from long‐term climate data that characterise typical rainfall patterns in each sampling unit in millimetres (National Computational Infrastructure 2012–2022). The human disturbance covariates included the Human Footprint Index (hereafter, “human footprint”) and the Forest Landscape Integrity Index (hereafter, “forest integrity”). Human footprint is a globally continuous measure that incorporates human population and infrastructure and is scored between 0 and 50 (Venter et al. 2016). Forest integrity is a geographic information system (GIS) layer that scores forest conditions between 0 and 10, based on both observed degradation (e.g., logging) and inferred degradation from edges and habitat connectivity (Grantham et al. 2020). Forest integrity does not distinguish forest types, only forest quality/integrity; thus, high‐integrity forests could be unsuitable for cassowaries if they are not rainforest (e.g., sclerophyll vegetation types).

To account for linear and non‐linear relationships, we evaluated human footprint and forest integrity using two modelling approaches. For our first approach, both covariates were treated as continuous predictors. All covariates were standardised (mean = 0, SD = 1) to place them on a comparable scale and improve model convergence. We examined Pearson correlation coefficients to ensure no collinearity among covariates (*r* < |0.5|). As a secondary approach, we grouped the covariate values into categories based on established thresholds, with adjustments to ensure adequate sample sizes across all categories. Human footprint was categorised as: No‐Low, Medium and High. Forest integrity was categorised as: “Low” (0–7) and “High” (8.9–10). We then created ordered categorical variables by applying orthogonal polynomial contrasts to human footprint (three levels) and forest integrity (two levels). In the main text, we present models using the human footprint covariate as a categorical predictor and forest integrity as a continuous variable because these performed better in AIC model selection. We include results from models using the continuous approach for human footprint and the categorical approach for forest integrity in the supplement (Table S6).

### 2.5 Royle–Nichols Modelling

We assessed cassowary detection probability, relative abundance, and habitat associations using the Royle–Nichols (RN) extension of basic hierarchical occupancy models (MacKenzie et al. 2002; Royle and Nichols 2003). We chose RN versus traditional occupancy models because RN performs well when there is very high occupancy throughout key areas of the range, as Westcott et al. (2014) suggested for cassowaries in tropical forests. The RN model uses presence–absence data to derive λ, a relative abundance metric, by exploiting the positive relationship between variation in individual detection probability and the species' abundance (Royle and Nichols 2003). RN models can reveal species‐habitat associations by estimating λ relative abundance along environmental or other covariate gradients while accounting for imperfect detection (MacKenzie et al. 2017; Sollmann 2018). We are conservative in interpreting the results from the RN model and avoid inferring that these values reflect the actual abundance or density. Rather, λ is only informative when being predicted by covariates to reflect spatial variation in relative abundance (Gilbert et al. 2021).

We ran single‐species, single‐season RN models in the unmarked R package (Fiske and Chandler 2011). To account for the nested hierarchical structure of sampling across different landscapes, we resampled nearby cameras into 3 km^2^ sampling units and included landscape as a fixed effect. We employed a stepwise model selection approach for our RN models, beginning with the detection process. Our null model only contained a landscape covariate in the abundance (state) formula. Then we ran univariate models for each detection covariate. Model comparisons were performed using the Conditional Akaike Information Criterion [AIC, (Vaida and Blanchard 2005)]. In cases where there were multiple competing univariate models (∆AIC < 6) and the covariates were not strongly correlated (*r* < |0.5|), we implemented multivariate models to determine the best combination of covariates. After determining the best detection formula, while still including the key covariate of trail status, we moved on to model selection for the abundance (state) formula, retaining the same top detection covariates. We used the same model selection procedure to assess covariates that should be included in the abundance (state) formula (Table 3).

**TABLE 3:** Model selection for detection and state covariates describing cassowary relative abundance in the tropical forests in northeast Australia.

	df	AIC	ΔAIC	ω
Detection formula				
Cameras active in sampling unit + trail status	139	1079.89	0.00	0.72
Cameras active in sampling unit	140	1081.75	1.87	0.28
Null	140	1133.51	53.63	0.00
State formula				
Forest integrity × rainfall	134	1060.34	0.00	0.65
Forest integrity + rainfall	135	1061.90	1.56	0.30
Human footprint categorical × rainfall	132	1066.67	6.33	0.03
Human footprint categorical + rainfall	134	1067.63	7.29	0.02
Human footprint categorical	135	1069.57	9.23	0.00
Rainfall	136	1070.86	10.52	0.00
Forest integrity	136	1075.26	14.93	0.00
Null	137	1079.89	19.55	0.00

*Note:* The table presents the degrees of freedom (df), corrected Akaike Information Criterion (AIC), the difference in AIC from the best‐fitting model and the top model (∆AIC) and the AIC weight (ω) for each model. Only models within 15 AIC points of the top model and the null model are included.

We started with analyses that incorporated all 10 landscapes within the cassowary range, including those with only small forest patches, those predicted to be unsuitable habitats (i.e., no rainforest) and those with no cassowary detections. However, the models including the unsuitable sites without cassowaries performed notably worse and produced unintuitive results (Tables S4 and S5). Therefore, we excluded two landscapes with unsuitable habitat and no cassowary detections. These were the Eacham‐Curtain Figure NP landscape, which is a collection of small and highly fragmented habitat patches with a combined area < 12 km^2^ and the Tumoulin NP, where cameras were primarily located outside of the rainforest. However, we retained one landscape with a large area of suitable rainforest but no cassowary detections (Paluma) because the species is known to persist there, and thus the absence of captures reflects imperfect detection. Therefore, in the main text, we report analysis from RN models that included eight tropical forest landscapes within the cassowary range and with suitable habitat (Table 2).

We included the number of cameras active in the sampling unit as both an observation‐level covariate and as an offset. The ecological inferences remained consistent across both approaches, but AIC model selection supported the offset formula (Tables S2 and S3); therefore, we retained it as an offset in the main text.

### 2.6 Mapping Relative Abundance

We used the top model identified through our model selection process to predict cassowary relative abundance across their tropical forest range in northeast Australia. Predictions were made across a 3 km^2^ cell raster to ensure consistency with the model. Predictions outside the range of observed values should be interpreted with caution due to increased uncertainty.

## 3 Results

**FIGURE 3:**
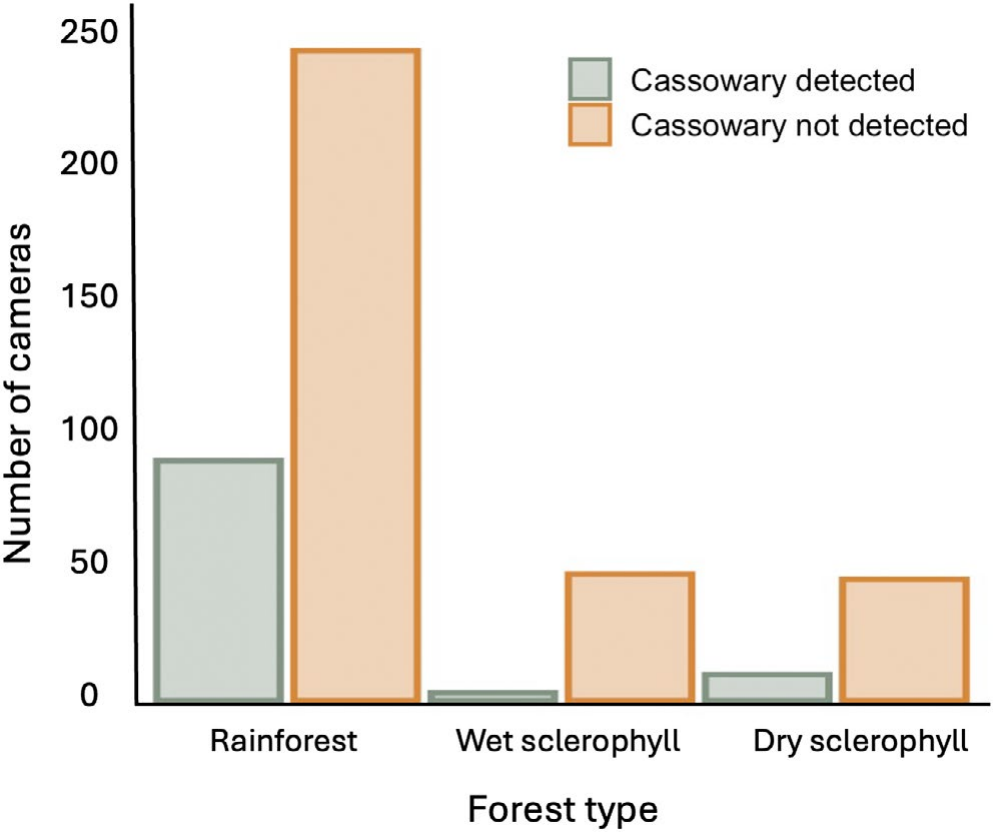
The number of cameras with cassowary detections versus non‐detections across three forest types: Rainforest, wet sclerophyll, and dry sclerophyll. Bars represent the total number of cameras deployed in each forest type, categorised by whether cassowaries were detected or not detected. A detailed map of forest type is provided in Figure S1.

There were 566 independent cassowary detections at seven of eight cassowary‐occupied landscapes during a total effort of 26,014 trap nights (Table 2). Wooroonooran NP recorded the most detections (293 during 4203 trap nights), and—among occupied landscapes—Kirrama NP had just three detections during 1619 trap nights and Paluma NP had no cassowary detections during 3342 trap nights (Table 2). There was a much lower percentage of cameras detecting cassowaries for cameras placed in wet and dry sclerophyll habitats compared to rainforests (Figure 3).

For the RN modelling of cassowary relative abundance, we first identified detection covariates through AIC model selection, with the number of active cameras and trail status retained in all subsequent RN models when assessing the best covariates for the state formula (details in Table S1). For the relative abundance (state) formula, there was strong support for models including forest integrity and rainfall as predictors of cassowary relative abundance (Table 3). The top model included an interaction between forest integrity and rainfall, with a competing additive model also receiving some support. Together, these models account for 94% of the AIC weight.

**FIGURE 4:**
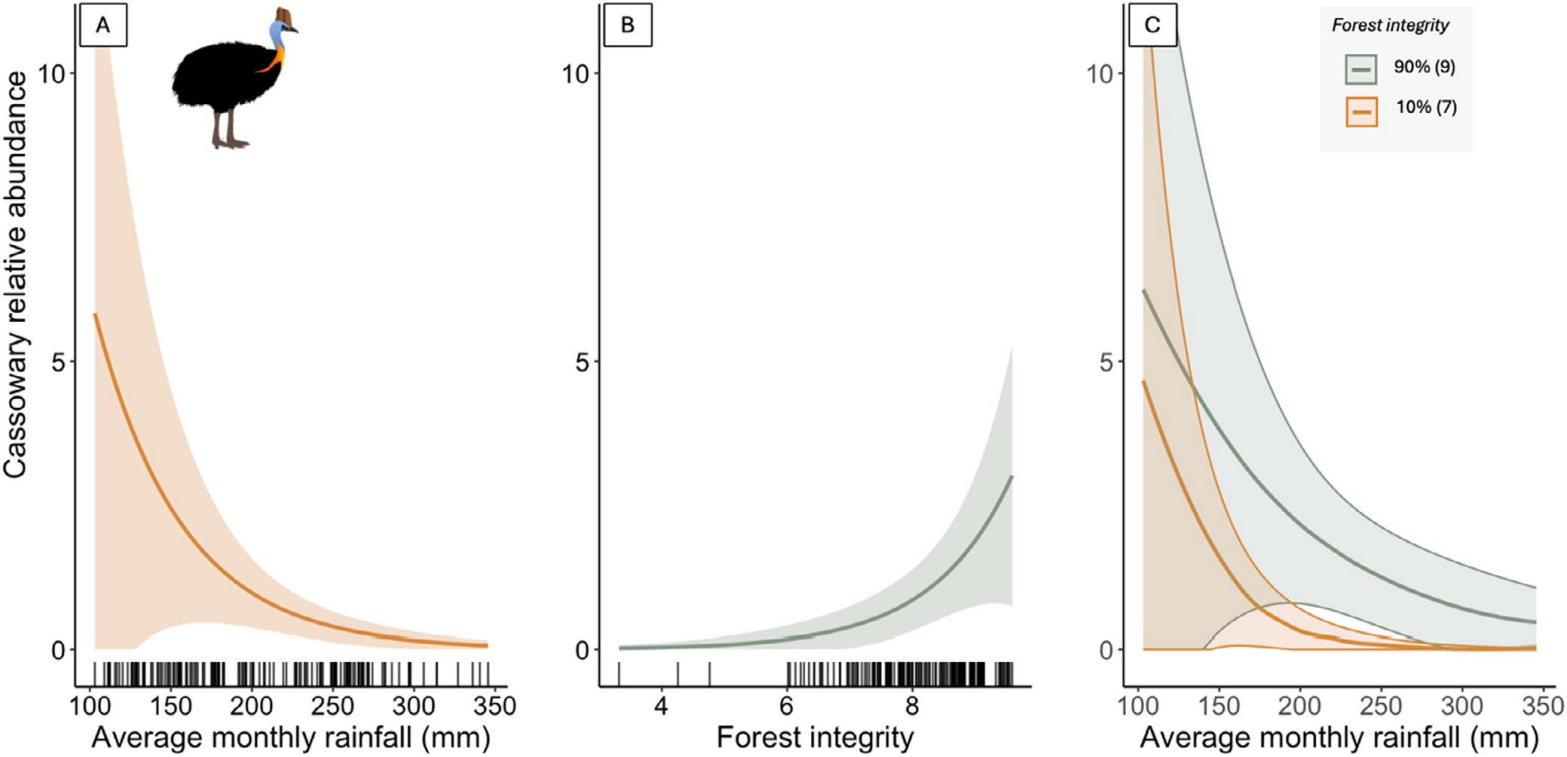
Predicted cassowary relative abundance (λ in RN hierarchical models) for the lowest‐scoring AIC model. Panels (A) and (B) depict the trend for each covariate while holding the other covariate constant at its mean value. Black tick marks along the *x*‐axis represent the distribution of the sampled covariate. Panel (C) illustrates the effect of rainfall on cassowary abundance while forest integrity is held at 10% (index value = 7) and 90% (index value = 9) of its maximum value. Shaded areas represent the 95% confidence intervals.

**FIGURE 5:**
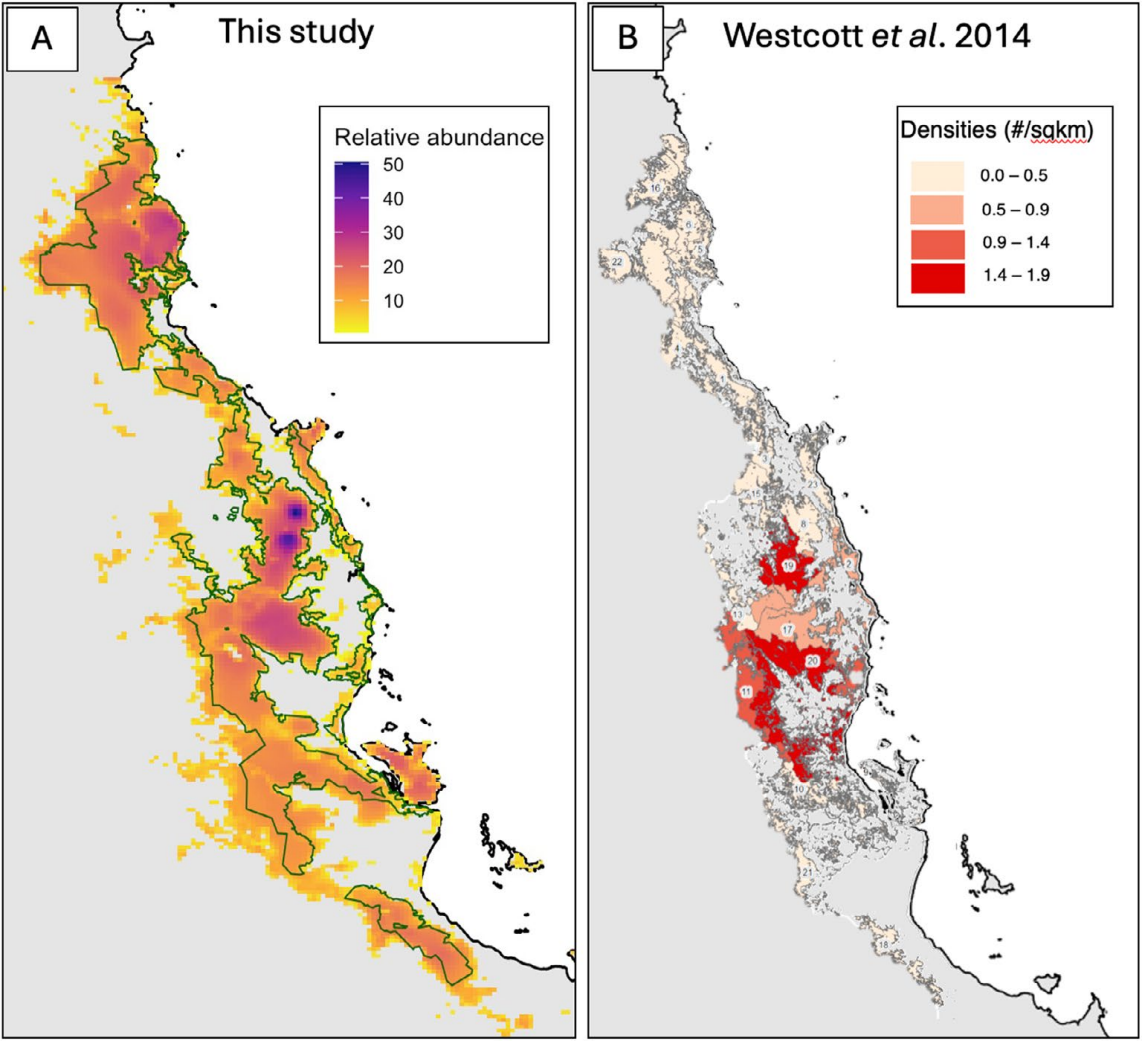
(A) Predicted cassowary relative abundance across Northeast Queensland, derived from the Royle–Nichols model, including an interaction between forest integrity and rainfall. (B) Cassowary density and distribution based on sign transects (redrawn from Westcott et al. 2014).

The top model suggested a significant positive association with forest integrity and a negative association with rainfall (coefficient Wald *z*‐tests were both *p* < 0.01; Table 4; Figure 4). There was also a marginally significant positive interaction between rainfall and forest integrity (coefficient Wald *z*‐test, *p* = 0.06), suggesting stronger cassowary avoidance of wetter‐degraded forest compared to drier‐degraded forest. We predicted and mapped cassowary relative abundance from the top model throughout their range, which showed hotspots in the core areas of the most extensive forests (Figure 5).

**TABLE 4:** Effect sizes, standard errors (SE), and *p*‐values of covariates affecting cassowary relative abundance from the top model.

	Estimate	SE	*z*	*p*
Detection fixed effects				
Intercept	−3.22	0.36	−9.00	0.00*
Trail status – on trail	−0.02	0.87	−0.03	0.98
Trail status – on trail/off trail	−0.89	0.38	−2.38	0.02*
State fixed effects				
Intercept	−0.10	0.32	−0.31	0.76
Forest integrity	0.85	0.26	3.24	0.00*
Rainfall	−1.15	0.28	−4.01	0.00*
Forest integrity × rainfall	0.46	0.24	1.90	0.06
Landscape fixed effects				
Daintree	−0.86	0.64	−1.34	0.18
Danbulla	0.84	0.47	1.80	0.07
Kirrama	−2.76	0.84	−3.27	0.00*
Koombooloomba	−0.59	0.52	−1.15	0.25
Mount Lewis	−1.93	0.62	−3.12	0.00*
Paluma	−0.46	0.5	−0.92	0.36
Wooroonooran Goldfield	−0.36	0.61	−0.59	0.56

*Note:* Trail status effects are shown relative to the base level of “off trail”, and landscape effects are shown relative to the base level of Wooroonooran National Park core. *p*‐values were obtained using Wald *z*‐tests (*z* = estimate/SE).

## 4 Discussion

This study is the first to use camera traps to investigate habitat associations of cassowaries across their Australian range in the Wet Tropics of Queensland. We found that cassowaries were rarely detected outside of rainforest habitat, and the strongest predictors of cassowary relative abundance were forest integrity and rainfall. Cassowaries were less abundant in fragmented forests and the wettest areas, and there was an interaction between these variables such that high forest integrity buffered the negative effect of increasing rainfall. This interaction is important, given climate change predictions of increased drought and rainfall variability in the region. The finding that cassowary relative abundance increased with forest integrity is consistent with findings from other Austral‐Asian tropical forest megafauna (Corlett 1998; Castelletta et al. 2000). When combined with the lack of significance for the human footprint covariate, our results suggest that cassowaries respond more to forest configuration (fragmentation) than to the presence of humans or what humans are doing adjacent to forests (urban versus farmland).

Rainfall ranges widely from 100 to 300 mm per month within the Wet Tropics, and cassowaries' relative abundance declined in the wettest areas (Queensland Government 2024). One reason is that our driest sites were still very wet, falling well within the criteria for a rainforest, while sites on the higher end of the rainfall spectrum include some of the wettest places on Earth. Thus, a more appropriate interpretation is that cassowaries may avoid areas with extreme rainfall peaks. Another reason is that there could be an unmeasured variable that correlates with rainfall, such as fruit availability. Studies in the Wet Tropics have shown that plant species producing cassowary‐attractive fruits increased flowering and fruiting in places and times when there was lower rainfall (Vogado et al. 2022, 2023). This may be due to limited photosynthetically active radiation to support fruit production when there is extreme cloud cover, such as the places and times with the most rainfall. Conversely, other research indicates that higher rainfall correlates with increased fruit productivity (Nepstad et al. 2002; Dunham et al. 2018). These contradictory relationships between rainfall and fruiting limit inferences for cassowaries and underscore how poorly phenological patterns are understood in tropical forests, especially in Australia (Pau et al. 2011). Wright (2005) found that dwarf cassowaries in Papua New Guinea adjusted their movement and diets in response to seasonal changes in fruit availability, reinforcing the view that cassowary abundance is tied to fruiting phenology, rather than rainfall.

Our findings about habitat associations and our predictive mapping provide a more nuanced understanding of the factors underpinning previously identified patterns in cassowary abundance and distribution observed during a sign‐based survey conducted a decade earlier (Westcott et al. 2014). Our mapping ignored park paper boundaries and used continuous mapping across ecological conditions by leveraging newly available high‐resolution GIS layers (Venter et al. 2016; Grantham et al. 2020). Despite using distinct sampling methods, our findings of cassowary abundance throughout the region broadly parallel Westcott's conclusions of high densities in the Atherton Tablelands (Figure 5). This provides confidence in our general understanding of cassowary distribution and ecology in Australia. Our map predicted high cassowary abundances, shown as dark purple hotspots, that directly coincide with Mount Belleden Ker and Mount Bartle Frere—two prominent mountains and the wettest parts of Australia. These regions receive average monthly rainfall of > 500 mm, substantially higher than the maximum rainfall observed in our sampling dataset (350 mm). As these areas represent ecological extremes outside the range of our sampling, predictions for these zones are likely unreliable and should be interpreted cautiously.

Westcott et al. (2014) also found low cassowary densities in the southern regions of the Wet Tropics, which they attributed to the impacts of Cyclone Yasi, given that cassowary signs and sightings were previously common in these areas. Almost a decade later, our sampling efforts also failed to detect cassowaries in the southern region of the Wet Tropics (Paluma National Park). However, our predictive map suggests that this region possesses a suitable habitat for cassowaries, supporting Westcott's conclusion that stochastic events like Cyclone Yasi may have severely impacted local populations, and they have yet to recover. Some have raised concerns that Paluma and the southern forested regions of the Wet Tropics are isolated from the rest of the rainforest by expanses of sclerophyll forests (Figure S1), making re‐colonization of the area unlikely. However, the lack of detections in Paluma is inconsistent with the most recent local knowledge that reports detections from Paluma (S.E. Anderson, personal observation). Therefore, additional camera monitoring of landscapes like Paluma will be necessary for tracking the recovery of this species in the south.

We did not use baits or lures in our camera trapping; however, cassowary detection rates may be improved using coloured lures at camera traps (McLean et al. 2017). We caution that lures have drawbacks, including altered (non‐random) movement patterns and variable attraction based on age, sex, or resident status (Gerber et al. 2011; Rovero and Zimmermann 2016; Dehaudt et al. 2024). Further, using baits limits the use for monitoring other species with the same cameras. Since the use of lures can have disadvantages, they are usually only advised for the most cryptic and least detectable species and if they significantly improve the precision of occupancy, abundance, or density estimates (Rovero and Zimmermann 2016). Cassowaries are not cryptic, and we found they are easily monitored with unbaited cameras.

Cassowaries' habitat associations did not support our initial hypothesis of a negative trend with the human footprint covariate (Kirika et al. 2008; Newbold et al. 2014). This may be due to some positive effects of humans, such as cassowaries being subsidised by cultivated fruit species that are common at forest edges and near residential areas (Wright 2005; Westcott et al. 2008). Similarly, recent research on Southeast Asian tropical forest‐dwelling frugivores shows a surprising tolerance to human‐modified landscapes (Dehaudt et al. 2022; Honda et al. 2023; Mendes et al. 2024). These findings point to either a degree of tolerance to human‐induced habitat conditions, altered biophysical conditions in forest edge effects and non‐forested areas (Luskin and Potts 2011), a lack of alternative habitat options (Luskin 2010; Pinondang et al. 2024), or that food subsidies at forest edges are bolstering disturbance‐tolerant frugivores, mesopredators, and generalists (Luskin et al. 2017, 2023; Moore et al. 2023). Given the historic deforestation, another possibility is that species and individuals most sensitive to anthropogenic disturbances may have already gone extinct. As a result, the remaining species and individuals may have passed through an extinction filter and are more resilient to these disturbances (Betts et al. 2019; Amir et al. 2022).

The findings suggest cassowaries are relatively common across Australian tropical rainforests, which is important for large‐seed dispersal and restoration. Cassowary seed dispersal improves habitat quality in corridors between forest fragments and supports the regeneration of degraded landscapes (Campbell et al. 2023). This is crucial because Australia's remaining tropical forests are globally significant, based on high endemism, evolutionary significance and phylogenetic distinctiveness (State of the Wet Tropics report 2022–2023). However, these forests are also highly fragmented and severely threatened by climate change (Williams et al. 2003; Campbell et al. 2023). The size of the cassowary population is tied to the availability and quality of remaining rainforest cover, making their reliance on the region's limited habitat a pressing concern that demands routine monitoring (Bruce et al. 2025).

Our findings on cassowaries have broader implications for understanding large‐bodied frugivores and terrestrial birds in various ecological contexts. Despite being habitat specialists, cassowaries demonstrated unexpected tolerance to human disturbances, challenging the assumption that habitat specialisation necessarily indicates disturbance intolerance (Colles et al. 2009; Clavel et al. 2010; Ockinger et al. 2010). Similar sampling could be conducted to investigate if this pattern extends to other large ratites like emus ( *Dromaius novaehollandiae* ), which occupy drier grassland environments in Australia. Beyond Australia, our results could improve our understanding of cassowary populations in Papua New Guinea, where different human pressures and habitat variables exist. Our results align with those of Honda et al. (2024), who found that—contrary to prior reports—binturongs ( *Arctictis binturong* ), a large, semi‐arboreal, frugivorous civet in Southeast Asia, were present in forest edges and degraded landscapes. These parallels suggest that some specialised rainforest frugivores may exhibit greater tolerance to humans than previously appreciated.

Future research on cassowaries should consider the effects of top‐down ecological regulation from predators, bottom‐up fruit availability, competition with invasive competitors, and long‐term population trends. Key species interactions include predation from dingos, competition from feral pigs, and domestic ungulates (multiple deer species and cows) that make their way into tropical forests (Taylor et al. 2011). Such analyses could use similar camera‐trapping datasets but apply Bayesian hierarchical N‐mixture co‐abundance models to explore the species interactions (Amir, Sovie, et al. 2022). Two other frontiers for cassowary ecology and conservation include their susceptibility to Avian influenza (Klaassen and Wille 2023; Wille et al. 2023) and how climate change will alter tropical forest habitats and their fruiting phenology that cassowaries depend on (Butt et al. 2015).

## Author Contributions

**Skye Elise Anderson:** conceptualization (equal), data curation (equal), formal analysis (equal), investigation (equal), methodology (equal), project administration (equal), visualization (equal), writing – original draft (equal), writing – review and editing (equal). **Zachary Amir:** conceptualization (equal), data curation (equal), formal analysis (equal), funding acquisition (equal), investigation (equal), methodology (equal), project administration (equal), supervision (equal), visualization (equal), writing – original draft (equal), writing – review and editing (equal). **Tom Bruce:** conceptualization (equal), data curation (equal), formal analysis (equal), funding acquisition (equal), investigation (equal), methodology (equal), project administration (equal), supervision (equal), visualization (equal), writing – original draft (equal), writing – review and editing (equal). **Matthew Scott Luskin:** conceptualization (equal), data curation (equal), formal analysis (equal), funding acquisition (equal), investigation (equal), methodology (equal), project administration (equal), supervision (equal), visualization (equal), writing – original draft (equal), writing – review and editing (equal).

## Conflicts of Interest

The authors declare no conflicts of interest.

## Supporting information


Data S1

## Data Availability

Data is available on figshare via this link doi: https://figshare.com/articles/dataset/Range‐wide_camera_trapping_to_reveal_cassowary_habitat_associations/28050704?file=51265058.
