# Supplementary material for: Range‐Wide Camera Trapping for the Australian Cassowary Reveals Habitat Associations With Rainfall and Forest Quality
Source: Ecol Evol. 2025 Jun 4;15(6):e71464. doi: 10.1002/ece3.71464 (PMC12134640; doi:10.1002/ece3.71464)
Supplement: Supplementary file 1 — Data S1 [file ECE3-15-e71464-s001.docx]

**Supplementary materials**

| Detection model | df | AIC | ΔAIC | ω |
| --- | --- | --- | --- | --- |
| Cameras active in sampling unit + trail status | 137 | 1079.89 | 0.00 | 0.72 |
| Cameras active in sampling unit | 140 | 1081.75 | 1.87 | 0.28 |
| Camera type | 124 | 1119.24 | 39.35 | 0.00 |
| Season | 137 | 1124.79 | 44.90 | 0.00 |
| Trail status | 137 | 1125.47 | 45.59 | 0.00 |
| Null | 140 | 1131.40 | 51.51 | 0.00 |
| Feature | 124 | 1132.00 | 52.11 | 0.00 |
| Source | 120 | 1133.51 | 53.63 | 0.00 |

Table S1. Full model selection table for detection variables describing cassowary relative abundance in tropical forests in Northeast Queensland. The table presents the degrees of freedom (df), corrected Akaike Information Criterion (cAIC), the difference in cAIC from the best-fitting model and the top model (∆AIC) and the AIC weight (ω) for each model.

Table S2. Detection and state model selection for cassowary relative abundance with camera effort treated as a fixed detection covariate. The table presents the degrees of freedom (df), corrected Akaike Information Criterion (cAIC), the difference in cAIC from the best-fitting model and the top model (∆AIC) and the AIC weight (ω) for each model.

| **Detection model** | **df** | **AIC** | **ΔAIC** | **ω** |
| --- | --- | --- | --- | --- |
| Cameras active in sampling unit + trail status | 136 | 1083.51 | 0.00 | 0.62 |
| Cameras active in sampling unit | 139 | 1084.50 | 0.99 | 0.38 |
| Camera type | 124 | 1119.24 | 35.73 | 0.00 |
| Feature | 137 | 1124.79 | 41.28 | 0.00 |
| Season | 137 | 1125.47 | 41.96 | 0.00 |
| Source | 140 | 1131.40 | 47.88 | 0.00 |
| Trail | 124 | 1132.00 | 48.49 | 0.00 |
| Null | 120 | 1133.51 | 50.00 | 0.00 |
| **State model** | **df** | **AIC** | **ΔAIC** | **ω** |
| Forest integrity * rainfall | 133 | 1065.08 | 0.00 | 0.59 |
| Forest integrity + rain | 134 | 1066.37 | 1.29 | 0.31 |
| Human footprint categorical * rainfall | 131 | 1070.20 | 5.12 | 0.05 |
| Human footprint categorical + rainfall | 133 | 1071.06 | 5.98 | 0.03 |
| Human footprint categorical | 134 | 1072.74 | 7.66 | 0.01 |
| Rainfall | 135 | 1074.83 | 9.75 | 0.00 |
| Forest integrity | 135 | 1079.24 | 14.16 | 0.00 |
| Null | 136 | 1083.51 | 18.44 | 0.00 |
| Elevation | 135 | 1085.46 | 20.38 | 0.00 |

Table S3. Effect sizes, standard errors (SE), and p-values of the forest integrity and rainfall interaction model for cassowary relative abundance when modelling camera effort as a fixed effect. Trail status effects are shown relative to the base level of ‘off trail’, and Landscapes are shown relative to the base level of Wooroonooran National Park.

| **Detection fixed effects** | **Estimate** | **SE** | **p-value** |
| --- | --- | --- | --- |
| Intercept | -1.93 | 0.39 | 0.00* |
| Cameras active in sampling unit | 0.08 | 0.01 | 0.00* |
| Trail status – off trail/on trail | -0.85 | 0.38 | 0.03* |
| Trail status – on trail | 0.06 | 0.88 | 0.94 |
| **State fixed effects** | **Estimate** | **SE** | **p-value** |
| Intercept | -0.15 | 0.32 | 0.64 |
| Forest integrity | 0.83 | 0.26 | 0.00* |
| Rainfall | -1.13 | 0.28 | 0.00* |
| Forest integrity * rainfall | 0.44 | 0.24 | 0.07 |
| **Landscape fixed effects** | **Estimate** | **SE** | **p-value** |
| Daintree | -0.78 | 0.65 | 0.23 |
| Danbulla | 0.92 | 0.47 | 0.06 |
| Kirrama | -2.62 | 0.84 | 0.00* |
| Koombooloomba | -0.50 | 0.52 | 0.34 |
| Mount Lewis | -1.85 | 0.62 | 0.00* |
| Paluma | -0.37 | 0.51 | 0.46 |
| Wooroonooran Goldfield | 0.44 | 0.24 | 0.07 |

Table S4. Model selection for cassowary relative abundance when all landscapes are included in the analysis. This supplementary analysis retains landscapes excluded in the main analysis (Eacham/Curtain Fig and Tumoulin). The table presents the degrees of freedom (df), corrected Akaike Information Criterion (cAIC), the difference in cAIC from the best-fitting model and the top model (∆AIC) and the AIC weight (ω) for each model.

| **Detection model** | **df** | | **AIC** | **ΔAIC** | **ω** |
| --- | --- | --- | --- | --- | --- |
| Cameras active in sampling unit + trail status | 151 | 1096.01 | | 0.00 | 0.72 |
| Cameras active in sampling unit | 154 | 1097.88 | | 1.86 | 0.28 |
| Camera type | 138 | 1138.49 | | 42.48 | 0.00 |
| Season | 151 | 1139.98 | | 43.96 | 0.00 |
| Feature | 151 | 1144.86 | | 48.84 | 0.00 |
| Trail status | 154 | 1150.09 | | 54.08 | 0.00 |
| Source | 134 | 1150.35 | | 54.34 | 0.00 |
| Null | 131 | 1151.01 | | 54.99 | 0.00 |
| **State model** | **df** | | **AIC** | **ΔAIC** | **ω** |
| Rainfall | 150 | 1076.92 | | 0.00 | 1.00 |
| Human footprint categorical | 149 | 1091.75 | | 14.82 | 0.00 |
| Forest integrity | 150 | 1095.10 | | 18.17 | 0.00 |
| Null | 151 | 1096.01 | | 19.09 | 0.00 |
| Elevation | 150 | 1097.30 | | 20.38 | 0.00 |

Table S5. Effect sizes, standard errors (SE), and p-values of the top model for cassowary relative abundance when all landscapes are included in the analysis. This supplementary analysis retains landscapes excluded in the main analysis (Eacham/Curtain Fig and Tumoulin. Trail status effects are shown relative to the base level of ‘off trail’, and Landscapes are shown relative to the base level of Wooroonooran National Park.

| **Detection fixed effects** | **Estimate** | **SE** | **p-value** |
| --- | --- | --- | --- |
| Intercept | -3.24 | 0.36 | 0.00* |
| Trail status – on trail | 0.19 | 0.71 | 0.78 |
| Trail status – off trail/on trail | -0.79 | 0.37 | 0.03* |
| **State fixed effects** | **Estimate** | **SE** | **p-value** |
| Intercept | 0.92 | 0.28 | 0.01* |
| Rainfall | -1.30 | 0.29 | 0.00* |
| **Landscape fixed effects** | **Estimate** | **SE** | **p-value** |
| Daintree | -2.79 | 1.02 | 0.01* |
| Danbulla | -2.43 | 0.65 | 0.00* |
| Eacham Curtain Fig | -1.00 | 0.55 | 0.07 |
| Kirrama | -4.05 | 0.86 | 0.00* |
| Koombooloomba | -1.71 | 0.54 | 0.00* |
| Mount Lewis | -3.07 | 0.67 | 0.00* |
| Paluma Range | -2.01 | 0.58 | 0.00* |
| Tumoulin | -2.90 | 0.75 | 0.00* |
| Wooroonooran Goldfield | -0.55 | 0.55 | 0.32 |

Table S6. Model selection for cassowary relative abundance in tropical forests of Northeast Queensland. This table presents the Forest Landscape Integrity Index as a categorical covariate and the Human Footprint Index as a continuous covariate, including the degrees of freedom (df), corrected Akaike Information Criterion (cAIC), the difference in cAIC from the best-fitting model and the top model (∆AIC) and the AIC weight (ω) for each model.

| **State model** | **df** | **cAIC** | **∆AIC** | **ω** |
| --- | --- | --- | --- | --- |
| Forest integrity categorical * rainfall | 134 | 1061.60 | 0.00 | 0.90 |
| Forest integrity categorical + rainfall | 135 | 1066.23 | 4.63 | 0.09 |
| Rainfall | 136 | 1070.86 | 9.26 | 0.01 |
| Forest integrity categorical | 136 | 1077.29 | 15.69 | 0.00 |
| Null | 136 | 1079.89 | 18.28 | 0.00 |
| Human footprint | 136 | 1081.74 | 20.14 | 0.00 |
| Elevation | 137 | 1081.82 | 20.22 | 0.00 |


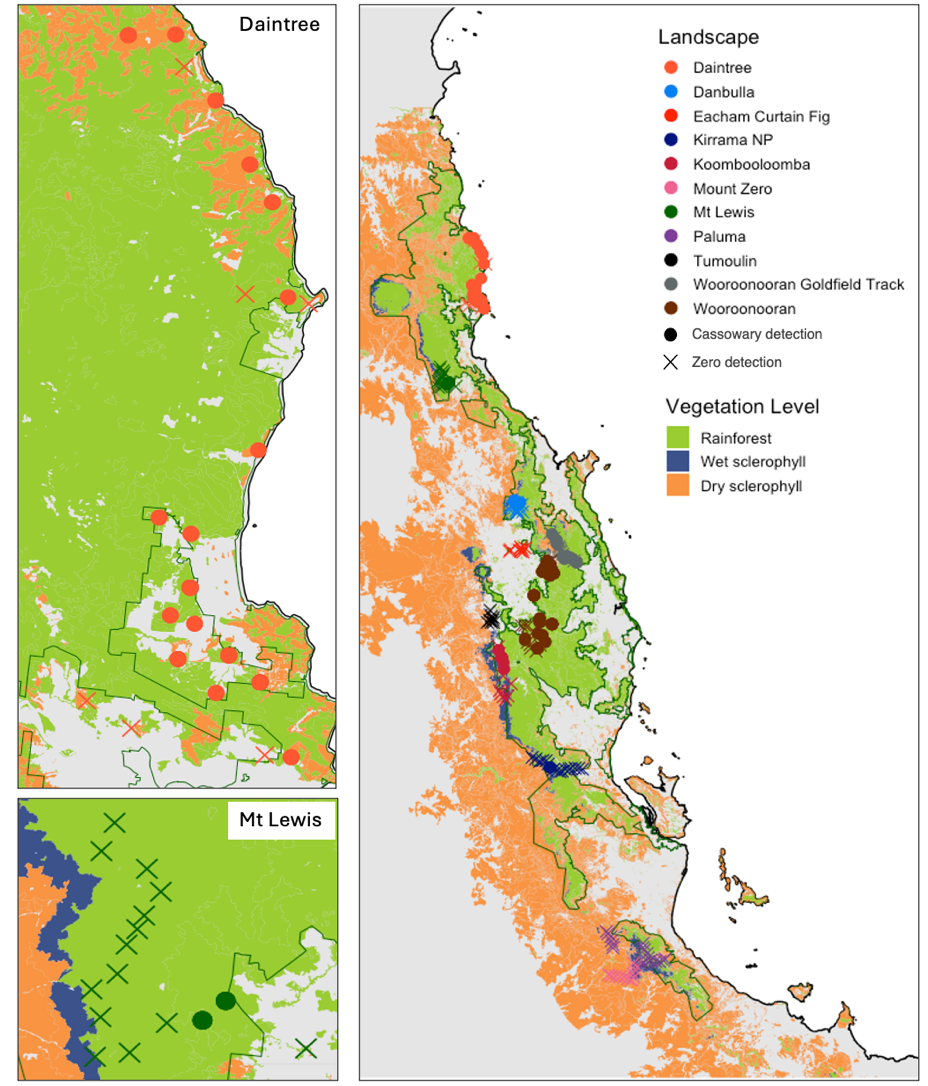


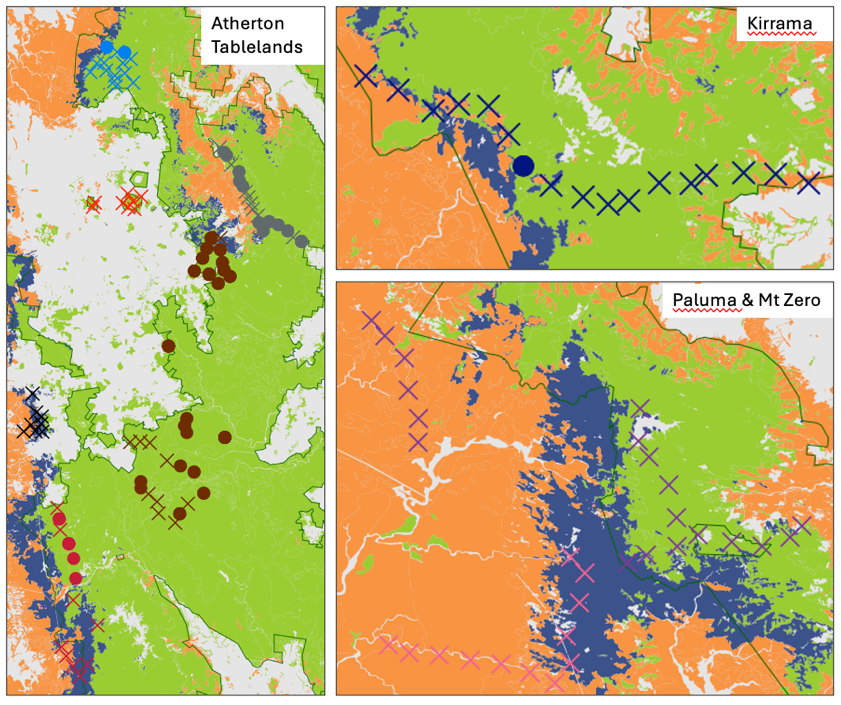


Figure S1. Map coloured by forest type (rainforest, wet sclerophyll and dry sclerophyll) across the cassowaries’ habitat range in Northeast Queensland, each point represents a sampling unit. Circles denote location where cassowaries were detected, while crosses indicate sites with no cassowary detections. Points are colour-coded by landscape.
